# Interleukin-18 binding protein protects against metabolic steatohepatitis

**DOI:** 10.1097/HC9.0000000000000840

**Published:** 2025-11-20

**Authors:** Emmanuel Somm, Yunju Jo, Elodie Perroud, Frédérique Ino, Karina Lindner, Baeki E. Kang, Christelle Veyrat-Durebex, Franck Bontems, Florian Visentin, Sébastien Fauteux-Daniel, Irmgard Förster, Anne-Claude Gavin, Sabrina Pagano, Nicolas Vuilleumier, Dongryeol Ryu, Karim Gariani, Cem Gabay, François R. Jornayvaz

**Affiliations:** 1 Service of Endocrinology, Diabetes and Metabolism, Department of Medicine, Geneva University Hospitals/University of Geneva, Geneva, Switzerland; 2 Diabetes Center, Faculty of Medicine, University of Geneva, Geneva, Switzerland; 3 Department of Cell Physiology and Metabolism, University of Geneva, Geneva, Switzerland; 4 Department of Biomedical Science and Engineering, Gwangju Institute of Science and Technology, Gwangju, Republic of Korea; 5 Molecular and Integrative Biology (MIB) Lab, Sungkyunkwan University School of Medicine, Suwon, Republic of Korea; 6 Department of Pathology and Immunology, Faculty of Medicine, University of Geneva, Geneva, Switzerland; 7 Immunology and Environment, Life and Medical Sciences (LIMES) Institute, University of Bonn, Bonn, Germany; 8 Division of Laboratory Medicine, Diagnostic Department, Geneva University Hospitals, Geneva, Switzerland; 9 Department of Medicine, Faculty of Medicine, Geneva University, Geneva, Switzerland

**Keywords:** fibrosis, inflammation, interleukin-18 binding protein, MASH, Western diet

## Abstract

**Background:**

Metabolic dysfunction–associated steatohepatitis (MASH) is a frequent consequence of Western diet consumption and liver steatosis. IL-18 binding protein (IL-18BP) limits the action of interleukin-18 (IL-18). Our work aims to study the unknown role of IL-18BP in MASH progression.

**Methods:**

We analyzed the liver transcriptome from MASH patients. We investigated cell-specific expressions of IL-18, IL-18BP, and IL-18 receptor in human and mouse liver. We studied the liver phenotype of *Il18bp* ^*−/−*^ mice on a high-fat/high-cholesterol (HFHC) diet. We administered an anti-IL-18 antibody in *Il18bp* ^*−/−*^ mice and in diet-induced wild-type (WT) MASH mice. We generated and studied double knock-out *Il18bp* ^*−/−*^ *Ifng* ^*−/−*^ mice.

**Results:**

IL-18BP expression is increased in the liver of patients and mouse models with MASH and positively correlates with fibrosis stages. On the HFHC diet, *Il18bp* ^*−/−*^ mice exhibit increased hepatic damage, inflammation, and fibrosis compared with WT mice. Treatment with anti-IL-18 antibody corrects liver defects in *Il18bp* ^*−/−*^ mice and ameliorates inflammation and fibrosis in diet-induced MASH mice, suggesting a translational treatment opportunity. Genetic deficiency in IFN-γ abrogates inflammation but not fibrosis in *Il18bp* ^*−/−*^ mice.

**Conclusions:**

IL-18BP has a role in limiting the progression of MASH, notably by reducing inflammation and fibrosis. Downstream IL-18 over-signaling, IFN-γ, mediates inflammation, but not fibrosis. Increasing IL-18BP levels represents a novel therapeutic perspective for patients affected by MASH.

## Graphical abstract

**Figure:**
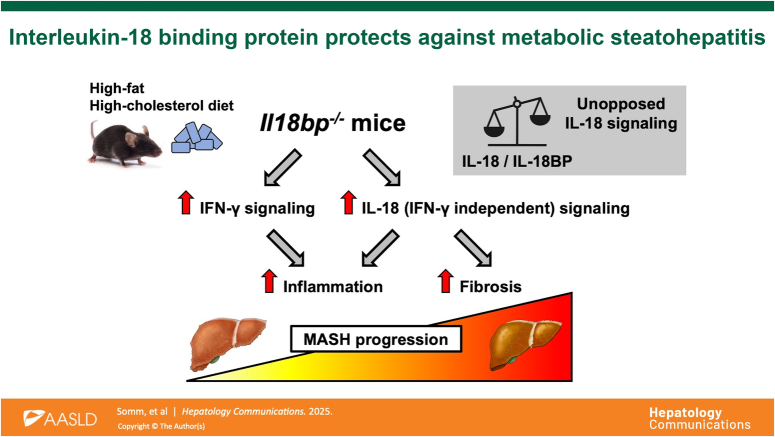

## INTRODUCTION

Metabolic dysfunction–associated steatotic liver disease (MASLD) is a growing public health concern, often caused by obesity and insulin resistance.^1^ A significant proportion of patients with MASLD experience a state of hepatic inflammation, now named metabolic dysfunction–associated steatohepatitis (MASH), which can result in hepatic fibrosis, cirrhosis, and hepatocellular carcinoma (HCC).^1^ While liver fat storage is quite reversible, fibrosis often represents a critical step in disease progression.^1^ Although lifestyle changes have beneficial effects, pharmacological treatments are still needed to treat MASLD/MASH.^1^

Interleukin-18 (IL-18) is a member of the IL-1 superfamily of cytokines.^2^,^3^ IL-18 precursor is processed by the inflammasome/caspase-1 complex into a mature/biologically active form.^2^,^3^ IL-18 triggers an IL-1-like intracellular signaling (ultimately leading to activation of NF-kB and inflammatory processes).^2^,^3^ Moreover, IL-18 specifically induces the production of interferon-gamma (IFN-γ), an action that explains its initial name of IFN-γ-inducing factor (IGIF). IL-18 exerts pleiotropic immune functions, including, for example, the expression of IL-2, IL-2 receptor, and Fas ligand expression on Th1 cells, or even the activation of natural killer cells,^2^,^3^ being in consequence a master regulator of innate and adaptive immunity.^2^,^3^

The role of IL-18 in inflammatory and infectious diseases is well established.^2^,^3^ Recent findings, in particular experimental studies involving genetically modified mice, also implicate IL-18 signaling in metabolism. Initially, IL-18-deficient (*Il18* ^*−/−*^) mice have been described as hyperphagic and obese, exhibiting secondary hepatic insulin resistance.^4^ Another study showed that *Il18* ^*−/−*^ mice develop hypercholesterolemia and hypertriglyceridemia before the manifestation of obesity, suggesting a primary liver defect.^5^ IL-18 receptor-deficient (*Il18r* ^*−/−*^) mice present a silencing of pro-inflammatory gene expression in MASLD, before the development of histologic MASH.^6^

The soluble IL-18 binding protein (IL-18BP) binds circulating IL-18 with high affinity, leaving only a small fraction of free IL-18 able to trigger receptor-mediated signaling.^2^,^3^ The role of IL-18BP in the context of MASLD/MASH has not yet been identified and is the main goal of the present study.

## METHODS

### Animals, diets, and treatments

All experimental protocols were performed in accordance with the Swiss animal welfare laws. Wild-type (WT) and *Il18bp* ^*−/−*^ male mice (generated as previously described^7^) on a pure C57BL/6 genetic background were housed in standard conditions in the animal facility of Geneva Medical Center. To generate the double *Il18bp* ^*−/−*^ *Ifng* ^−/−^ mice, *Il18bp* ^*−/−*^ mice were crossed with *Ifng* ^−/−^ mice (https://www.jax.org/strain/002287). Starting at 10 weeks of age, mice continued a chow diet (CHOW) (Safe150) or, to model MASLD/MASH, were fed with either a high-fat/high-cholesterol (HFHC) “atherogenic” diet (Research Diets#D17052505) or a choline-deficient amino acid-defined high-fat diet (CDAHFD) (Research diet#A06071302). HFHC diet potentiates the oxidative stress^8^ while CDAHFD promotes steatosis/inflammation/fibrosis more rapidly and potently.^9^ HFHC was provided during 14 weeks (initial phenotyping, Figures 2, 3) or during 6 weeks (antibody administration, Figure 4), while CDAHFD was provided during 3 weeks (antibody administration, Figure 5) or during 6 weeks (comparison of WT/*Il18bp* ^*−/−*^/*Il18bp* ^*−/−*^ *Ifng* ^−/−^ mice, Figure 6). For treatment with anti-IL-18 antibody, mice were injected i.p. 3 times per week with either saline (NaCl 0.9%), 0.5 mg/mouse of IgG1 isotype control (A2106, Selleckchem), or 0.5 mg/mouse of neutralizing mouse anti-mouse IL-18 mAb (clone SK113AE-4) generated as previously described.^10^ *Il18bp* ^*−/−*^ were treated during the 6 weeks of HFHC consumption (Figure 4) while WT C57BL/6J mice were treated during the 3 weeks of CDAHFD consumption (Figure 5). Double *Il18bp* ^*−/−*^ *Ifng* ^−/−^ mice were studied at 20 weeks of age after only chow diet consumption (Supplemental Figure S5, http://links.lww.com/HC9/C166) or after 6 weeks of CDAHFD consumption (Figure 6). In all experiments, only male mice of similar age were analyzed, allowing for the avoidance of the confounding impact of aging and hormonal variations. This strategy does not allow for elucidation of whether results/interpretations are applicable to female mice. At the end of all experiments, mice were fasted for 3 hours, slightly anesthetized with isoflurane, and immediately sacrificed. Blood samples were collected in EDTA-coated tubes/stored at −80 °C, and organs were dissected and weighed before fixation/cryopreservation in liquid nitrogen.

### Blood and tissue biochemical analyses

Plasma levels of ALT and AST were assessed using a Cobas C111 robot and supplied reagents (Roche Diagnostics). For hepatic triglyceride and cholesterol content, total lipids were extracted using methyl tert-butyl ether before quantification using a Cobas C111 robot. Liver collagen content was determined using a sensitive tissue collagen assay (QuickZyme-Biosciences). Blood and liver cytokine content were measured using the mouse V-Plex Proinflammatory Panel kit and the QuickPlex MSD SQ120 instrument from MesoScale Discovery (MSD, Rockville, MD, USA), following the manufacturer’s instructions.

### Gene expression

Total RNA isolated from liver samples using TRI Reagent Solution (Thermo Fisher Scientific) was reverse-transcribed using the MMLV kit (Invitrogen). cDNAs were quantified by real-time PCR using Power SYBR Green mix and a Light-Cycler 480 Detection System (Roche Diagnostics), normalized using the housekeeping gene Rps29, folded to the mean value of the corresponding control group, and expressed as arbitrary units (A.U.).

### Immunoblotting (western blot)

Human healthy and MASLD/MASH liver samples provided by LifeNetHealth (http://www.LifeNetHealth.org, Virginia Beach, VA, USA) were used to extract proteins using RIPA buffer (Thermo Fisher Scientific) and were subjected to reducing SDS–PAGE using 4%–12% Tris gels NuPAGE (Invitrogen). Proteins were then electroblotted from the gels on Nitrocellulose membranes (Amersham Hybond, GE Healthcare, Glattbrugg) and probed with the validated IL-18BP primary antibody (1:1000) and the housekeeping Vinculin antibody (1:1000), followed by anti-human horseradish peroxidase-conjugated secondary antibody. Enhanced chemiluminescence (ECL) mediated by horseradish peroxidase was revealed with the ECL kit (Amersham ECL Plus Western Blotting Detection Reagents, GE Healthcare, Glattbrugg) and detected with a FUSION FX machine (Vilber, Marne-la-Vallée, France).

### Histology/immunohistochemistry

For histology, livers were either fixed overnight in 10% formalin before dehydration and embedded in paraffin or immediately embedded in OCT medium (Cell Path Ltd) and frozen on dry ice before storage at −80 °C. Paraffin-embedded sections of liver were stained with hematoxylin–eosin (H&E) or Sirius Red (SR) using classical procedures. Frozen liver sections were stained with Oil Red O (ORO) using classical procedures. For immunohistochemistry, paraffin sections were dewaxed and rehydrated using xylene/ethanol baths and then heated at 95 °C in a 10 mM/pH 6.0 sodium citrate bath for 10 minutes. The liver sections were then incubated overnight at 4 °C with primary antibodies diluted in phosphate-buffered saline (PBS)/0.1% BSA, washed in PBS, and incubated for 1 hour with a secondary antibody (Alexa Fluor 488 goat anti-mouse) diluted (1:1000) in PBS/0.1% bovine serum albumin (BSA) (immunofluorescence) or revealed with the diaminobenzidine tetrahydrochloride (DAB)/horseradish peroxidase (HRP) system (90 seconds). The primary antibodies used were directed against mouse IBA1 (Wako 019-19741) (1:500), P-IKK^αβ^ (Cell Signaling Technology Cat# 2697) (1:200), NFKB (Cell Signaling Technology Cat# 8242) (1:1600), αSMA (Invitrogen MA5-11547) (1:200), and PDGFRb (Invitrogen MA5-15143) (1:200). Pictures were acquired using a VS120 microscope (Olympus) or an Axio Scan.Z1 slide scanner (Zeiss) or an Axiophot microscope/an Axiocam color camera (Zeiss). For histomorphometry, SR staining (fibrosis) was evaluated in 4 representative images per animal using the ImageJ software. The number of inflammatory foci per 200× field was counted manually, in 4 independent fields per animal, as clinically determined to evaluate the inflammatory component of the NAFLD activity score (NAS). ORO staining (neutral lipid content), NFKB, P-IKK^αβ^, and αSMA were quantified using the ImageJ software.

### Transcriptomic analyses

For bulk RNAseq related to MASLD/MASH patients, raw data originate from the publicly available GEO database GSE135251 (https://www.ncbi.nlm.nih.gov/geo/). Detailed phenotypic descriptions and demographics were reported previously, and the cohort was stratified according to NAS and fibrosis score.^11^ The expression value of each gene in the 2 disease states and the control group was compared using a Wilcoxon 1-sided test, and the results were visualized in boxplots (median±quartiles). The Kruskal–Wallis test was used to evaluate gene expression at the parameter level, and the results were presented as a *p-*value. Correlation matrices were generated based on the Spearman correlation coefficient rho. All computations were conducted using the R program (RStudio 1.4).

For bulk RNAseq related to mice, total RNA was isolated from the livers of 5 WT and 5 *Il18bp* ^*−/−*^ mice fed with HFHC diet for 14 weeks using TRI Reagent Solution (Thermo Fisher Scientific). After integrity control, sequencing was done on an Illumina NovaSeq. 6000 at the iGE3 Genomics Platform of Geneva University. The RNA-seq data were processed and analyzed using R (ver. 4.3.2) and RStudio (ver. 2023.03.0 Build 386). Differentially expressed gene (DEG) analysis was performed using the R package, DESeq (ver. 1.42.0). Adjusted *p*-values for each group comparison were calculated using the Benjamini–Hochberg method. Gene Set Enrichment Analysis (GSEA) was conducted using the GSEA Java desktop application from the Broad Institute (GSEA ver. 4.1.0 for Windows, https://www.gsea-msigdb.org). *p*-values were calculated using the Fisher exact test, with a significance cutoff of 0.05. The gene lists for each biological pathway were retrieved from the Molecular Signature Database. Depth of shading in the correlation matrices (correlogram) indicates the magnitude of the correlation (Spearman *r* *ho*). Correlogram and interaction network were generated using RStudio. Data preprocessing and visualizations, including bubble plots and heatmaps, were generated using the R packages *ggplot2* (ver. 3.5.0), *scales* (ver. 1.3.0), *viridis* (ver.0.6.4), *dplyr* (ver. 1.1.4), *tidyr* (ver.2.1.0), *reshape2* (ver.1.4.4), *egg* (ver.0.4.5), *RColorBrewer* (ver.1.1.3), and *pheatmap* (ver.1.0.12).

For scRNAseq, raw data originate from the publicly available databases and analysis tool, Protein Atlas (proteinatlas.org) and Liver Cell Atlas (livercellatlas.org). Uniform Manifold Approximation and Projection (UMAP) from the Protein Atlas originates from 8439 cells (44.1 M reads), while UMAP projection from the Liver Cell Atlas originates from 82,800 cells (mouse standard) and 68,600 cells (mouse MASLD).^12^,^13^

### Flow cytometry

For FACS analysis, livers were perfused in vivo with collagenase type 4 (0.1%) and DNase type I (0.005%) for 30 minutes before organ harvest. Organs were then crushed on a 50 μm strainer, and lymphocytes were isolated at the interface of 40%/80% Percoll density gradient (Cytiva). RBC lysis was then performed, and cells were blocked for CD16/CD32 for 15 minutes before surface staining for 30 minutes at 4 °C. Cells were then identified with the following definition: Myeloid cells were gated on CD45^+^NK1.1^−^CD11c^−^CD11b^+^ and further stratified into Ly6C^+/−^. T cells were identified as CD45^+^CD11b^−^CD19^−^CD117^−^Ly6G^−^CD3^+^ and subclassified into CD4^+^ or CD8^+^. Features of FACS parameters are listed in the dedicated table in the Supplemental Material, http://links.lww.com/HC9/C167.

### Statistical analyses

Statistical analyses of the data using one-way ANOVA or the Student *t* test were performed using the GraphPad Prism software. Bars represent mean ± SEM. A *p*-value <0.05 was considered statistically significant.

## RESULTS

### Liver IL-18BP expression is increased in MASLD/MASH patients, correlating with disease severity

**FIGURE 1:**
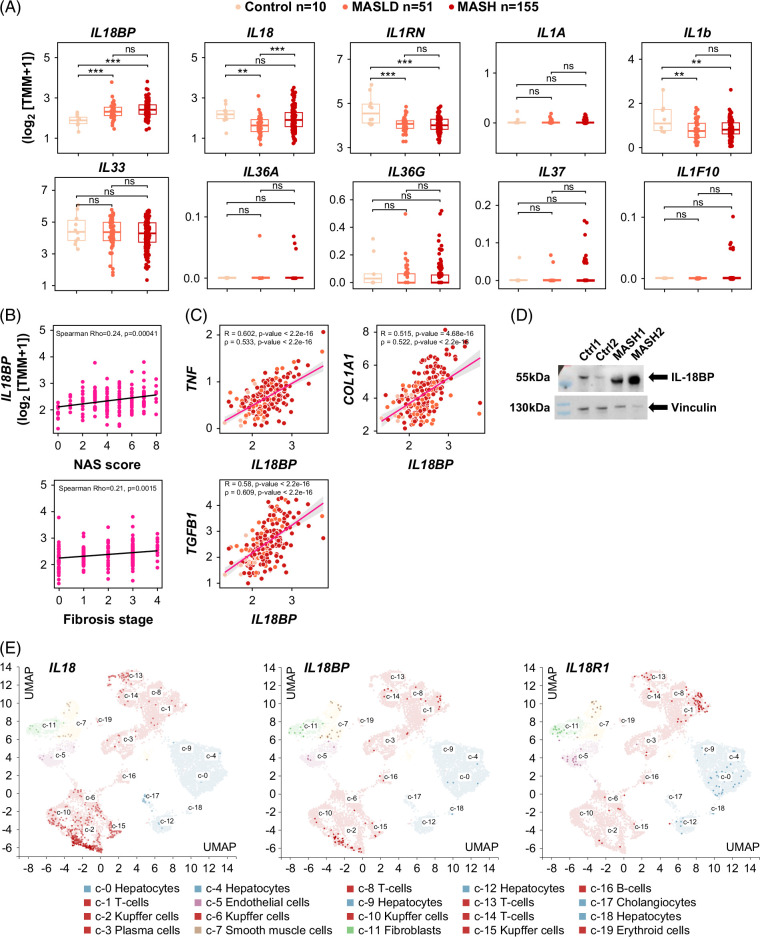
Hepatic IL-18BP is induced in MASLD/MASH patients. (A) Hepatic gene expression of IL-1 superfamily inhibitors and ligands in MASLD/MASH patients. (B) Hepatic *IL18BP* expression in MASLD/MASH patients as related to NAFLD activity score (NAS) or fibrosis stage. (C) Correlation between hepatic *IL18BP* expression levels in MASLD/MASH patients as related to hepatic *TNFA*, *COL1A1*, and *TGFB1* expression levels. (D) Immunoblot of IL-18BP on liver protein extracts of control and MASLD/MASH patients. (E) UMAP projection of *IL18*, *IL18BP*, and *IL18R1* in cell types composing the human liver. Raw data originate from the GEO database GSE135251 (A, B) and from the Human Protein Atlas (proteinatlas.org) (E). *p-*values calculated using the Kruskal–Wallis test or 1-tailed Wilcoxon rank sum test. *p<0.05, **p<0.01, and ***p<0.001 versus controls. Abbreviations: GEO, Gene Expression Omnibus; IL-18, interleukin-18; IL-18BP, interleukin-18 binding protein; MASH, metabolic dysfunction–associated steatohepatitis; MASLD, metabolic dysfunction–associated steatotic liver disease; NAS, NAFLD activity score; UMAP, Uniform Manifold Approximation and Projection.

Using the GSE135251 transcriptomic dataset, we observed that hepatic IL18BP expression (in contrast to other IL-1 superfamily members) was increased in patients with MASLD/MASH (Figure 1A), positively correlating with fibrosis stages and MASLD activity score (NAS) (Figure 1B), as well as with gene expression of pro-inflammatory and pro-fibrotic markers (Figure 1C). Accordingly, hepatic IL-18BP protein was also upregulated in patients with MASLD/MASH (Figure 1D). Single-cell RNA sequencing (scRNAseq) from the Human Gene Atlas database (Figure 1E) and from the Liver Cell Atlas database (Supplemental Figure S1, http://links.lww.com/HC9/C166) evidenced a predominant expression of IL18 and IL18BP in macrophage populations (in particular in Kupffer cells) and a main expression of IL-18 receptor (IL18R1) in T-cells/natural killer T (NTK) cells/endothelial cells/fibroblasts. Additional murine scRNAseq analysis corroborated human observations, confirming that *Il18bp* is predominantly expressed in Kupffer cells, while *Il18r1* is mainly expressed in type 1 innate lymphoid cells (ILC1s), natural killer cells (NK cells), different populations of T-cells [NTK cells, γδ T-cells, T-regulatory (TRegs) cells], and mesothelial cells in mice (Supplemental Figures S2–S4, http://links.lww.com/HC9/C166).

### IL-18BP deficiency exacerbates hepatic inflammation and fibrosis in mice on a Western diet

In line with clinical transcriptomic observations, *Il18bp* is overexpressed in the liver of both dietary and genetic mouse models exhibiting MASLD/MASH (Figure 2A). To unravel the role of IL-18BP in the disease progression, we analyzed the liver of IL-18BP-deficient (*Il18bp* ^*−/−*^) mice in basal condition (chow diet) and on a Western diet (HFHC diet). On chow diet, *Il18bp* ^*−/−*^ mice exhibited similar liver weight, transaminase levels, and liver histology compared with WT mice (Supplemental Figures S5B–D, http://links.lww.com/HC9/C166). While *Il18* and *Ifng* were overexpressed in the liver of *Il18bp* ^*−/−*^ mice on a chow diet (Supplemental Figure S5E, http://links.lww.com/HC9/C166), reflecting their unopposed IL-18 signaling, these changes were not associated with modifications in *Il1b* or *Tnfa* mRNA levels (Supplemental Figure S5E, http://links.lww.com/HC9/C166) or the appearance of inflammatory foci (data not shown). Together, these observations refute a spontaneous inflammation in the liver of *Il18bp* ^*−/−*^ mice. On the HFHC diet, *Il18bp* ^*−/−*^ mice exhibited increased relative liver weight (Figures 2C, D) and increased circulating ALT and AST levels (Figure 2E) compared with WT mice. Sirius Red and Oil Red O staining, collagen/triglyceride/cholesterol content, as well as measurement of pro-fibrotic markers, confirmed an aggravated liver fibrosis occurring independently of any changes in liver steatosis in *Il18bp* ^*−/−*^ versus WT mice (Figures 2F–K). Inflammatory foci proportion (Figure 2L) and immunolabelling of the macrophagic IBA1 marker (Figure 2F) reflect an exacerbated diet-induced hepatic inflammation in *Il18bp* ^*−/−*^ compared with WT mice. This pro-inflammatory phenotype was also supported by increased p-IKK^αβ^ staining and NF-κB staining in the liver of *Il18bp* ^*−/−*^ versus WT mice (Figures 2F, M).

FIGURE 2IL-18BP deficiency worsens hepatic inflammation and fibrosis in mice on an HFHC diet. (A) Hepatic gene expression of *Il18bp* in genetic (*db*/*db* mice) and dietary MASLD/MASH mouse models [high-fat diet (HFD), methionine and choline-deficient (MCD) diet, and high-fat/high-cholesterol (HFHC) diet]. (B) Schematic representation of the study protocol. (C) Images of livers. (D) Relative liver weight. (E) Circulating transaminase levels. (F) Sirius Red (SR), hematoxylin–eosin (H&E), Oil Red O (ORO) staining, and IBA1, p-IKK^αβ^, NF-κB immunostaining of liver sections. (G) SR positive staining. (H) Liver collagen content. (I) Liver gene expression of pro-fibrogenic markers. (J) ORO positive staining. (K) Liver triglyceride and cholesterol content. (L) Number of inflammatory foci per field (200×). (M) p-IKK^αβ^ and NF-κB positive staining. (N–Q) Fluorescence-Activated Cell Sorting analysis for myeloid and lymphoid cell lineages on liver non-parenchymal cells. Concerning panel A, 10-week-old male mice were fed either a chow diet (Safe 150) for 10 weeks, or a high-fat diet (HFD) (60 kcal%fat) for 10 weeks, or a methionine choline-deficient (MCD) diet for 7 weeks. Bars represent mean±SEM of individual values (circles). *p<0.05 versus WT mice (Student *t* test). n=4–7 male mice per group. Abbreviations: HFD, high-fat diet; HFHC, high-fat/high-cholesterol; IL-18BP, interleukin-18 binding protein; MASH, metabolic dysfunction–associated steatohepatitis; MASLD, metabolic dysfunction–associated steatotic liver disease; WT, wild type.

**Figure:**
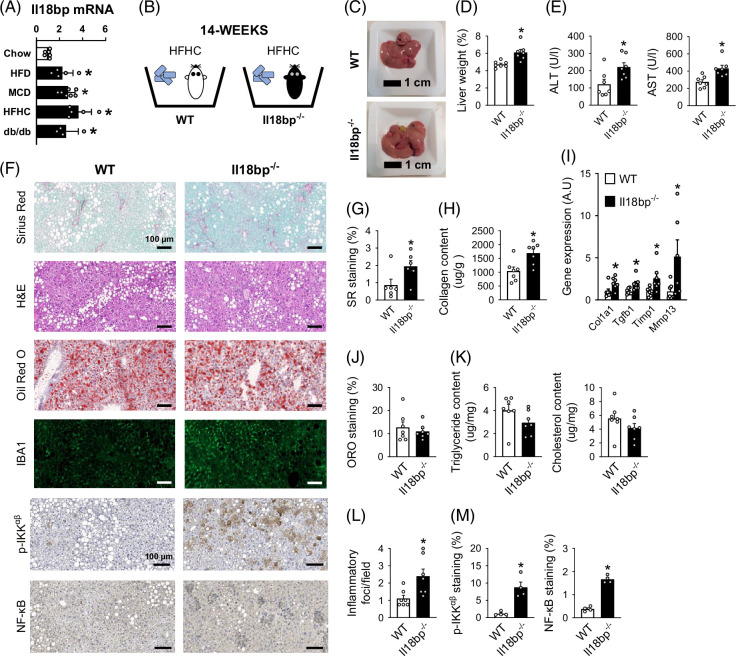

**Figure:**
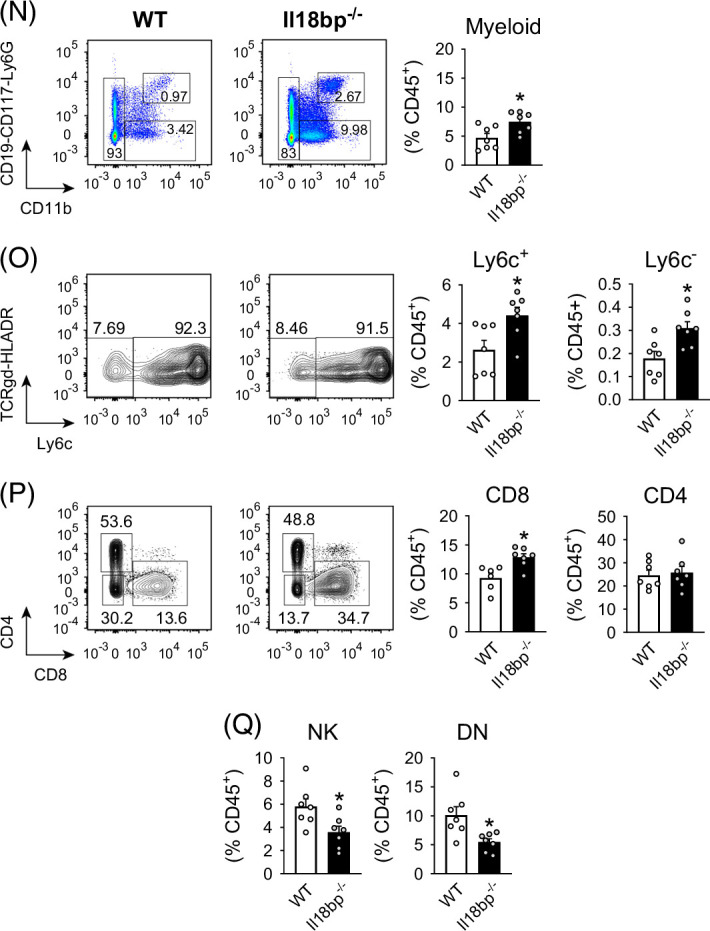

We deepened the immune-phenotyping of *Il18bp* ^*−/−*^ mice livers on HFHC diet using fluorescence-activated cell sorting (FACS) and transcriptomic approaches. FACS analysis revealed that *Il18bp* ^*−/−*^ mice livers present a significant enrichment in myeloid cells, in both recruited (Ly6c^+^) and resident (Ly6c^−^) macrophages as well as in CD8^+^ T cells (Figures 2N–P). In addition, *Il18bp* ^*−/−*^ mice livers present a significant reduction in natural killer (NK) cells, as well as in double negative T lymphocytes, when compared with wild-type mice livers (Figure 2Q).

In RNAseq experiments, Differential Gene Expression analysis (DGE) and Gene Set Enrichment Analysis (GSEA) confirmed that transcripts significantly upregulated in *Il18bp* ^*−/−*^ mice livers were enriched for gene ontology clusters related to inflammation and fibrosis (Figures 3A–D), substantiating their MASH-prone status. These results were corroborated by a wide targeted screening of mRNAs coding for inflammatory/immune markers through real-time quantitative PCR (qPCR). Gene expression of pro-inflammatory interleukins *Il1b*, *Il12*, *Tnfa*, as well as *Ifng* and downstream target chemokines (*Ciita*, *Cxcl9*, *Cxcl10*) were all upregulated in the liver of *Il18bp* ^*−/−*^ versus WT mice (Figure 3E). In line, liver protein content in TNF-α, IFN-γ, and IL-1β, as well as circulating levels of TNF-α and IFN-γ, were also increased in *Il18bp* ^*−/−*^ compared with WT mice on HFHC diet (Figures 3I, J). mRNA levels of blood-derived macrophages (*Ly6c*/*Ccr2*) and dendritic cells (*Mhc2*/*Cd103*) markers were also all upregulated in *Il18bp* ^*−/−*^ mice livers (Figure 3F). In contrast, the expression of the phosphatidylserine receptor Timd4, a specific marker of mature KC, was reduced by half in *Il18bp* ^*−/−*^ versus WT mice livers (Figure 3F), as previously observed in the context of MASH progression/worsening.^14^,^15^,^16^ More surprisingly, a concomitant overexpression of both type 1 (*Cd14*/*Nos2*) and type 2 (*Il5*/*Il10*/*Il13*) immunity markers was also observed in nutritionally challenged *Il18bp* ^*−/−*^ mice livers (Figure 3G). Interestingly, hepatic gene expressions of *Il10* and *Il13* correlated with collagen content (Figure 3K). Finally, gene expression of enzymes involved in bile acid synthesis (*Cyp8b1*/*Cyp7b1*/*Hsd3b7*) was drastically reduced, while that of the enzyme involved in 25-Hydroxycholesterol synthesis (*Ch25h*) was significantly increased in the liver of *Il18bp* ^*−/−*^ versus WT mice (Figures 3H, L).

FIGURE 3Transcriptomic analysis underlying worsened hepatic inflammation in IL-18BP-deficient mice on high-fat/high-cholesterol (HFHC) diet. (A) Heat map and volcano plots showing differentially expressed genes. (B) Enrichment plots. (C) Annotated heat map. (D) Pathway networks recapitulating GSEA. (E) Liver gene expression of pro-inflammatory cytokines/chemokines. (F) Liver gene expression of macrophage and dendritic cell markers. (G) Liver gene expression of immune polarity markers. (H) Liver gene expression of enzymes involved in bile acids/oxysterols synthesis. (I) Liver cytokine protein content. (J) Blood cytokine protein content. (K) Correlations between liver gene expression of IL-10 or IL-13 and liver collagen content. (L) Pathways of bile acids/oxysterols synthesis. Genes indicated in red are downregulated, while genes indicated in green are upregulated in *Il18bp* ^*−/−*^ versus WT mice. Bars represent mean±SEM of individual values (circles). *p<0.05 versus WT mice (Student *t* test). n=5–7 male mice per group. Abbreviations: A.U., arbitrary unit; GSEA, Gene Set Enrichment Analysis; IL-18BP, interleukin-18 binding protein; NK, natural killer; WT, wild type.

**Figure:**
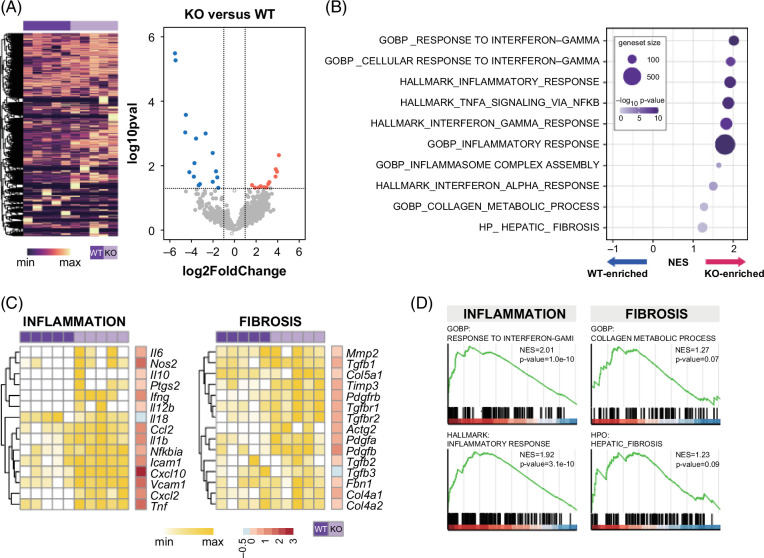

**Figure:**
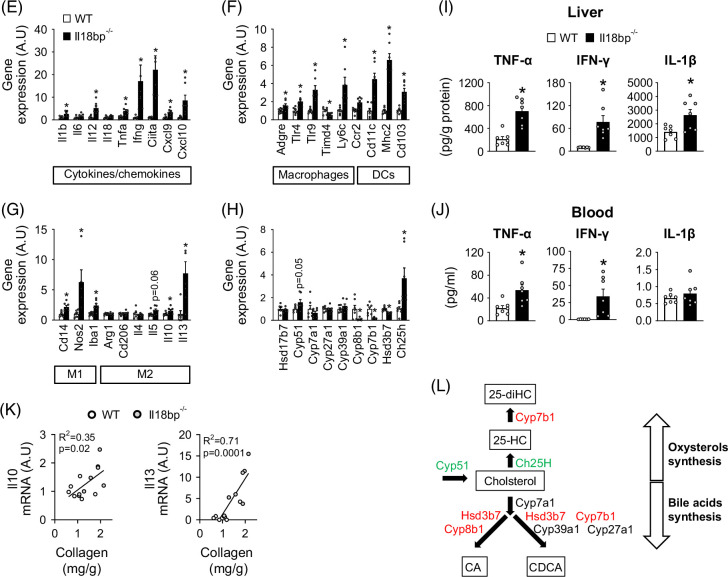

### IL-18 blockade attenuates liver defects in *Il18bp* ^*−/−*^ mice as well as in WT MASLD/MASH mice

**FIGURE 4:**
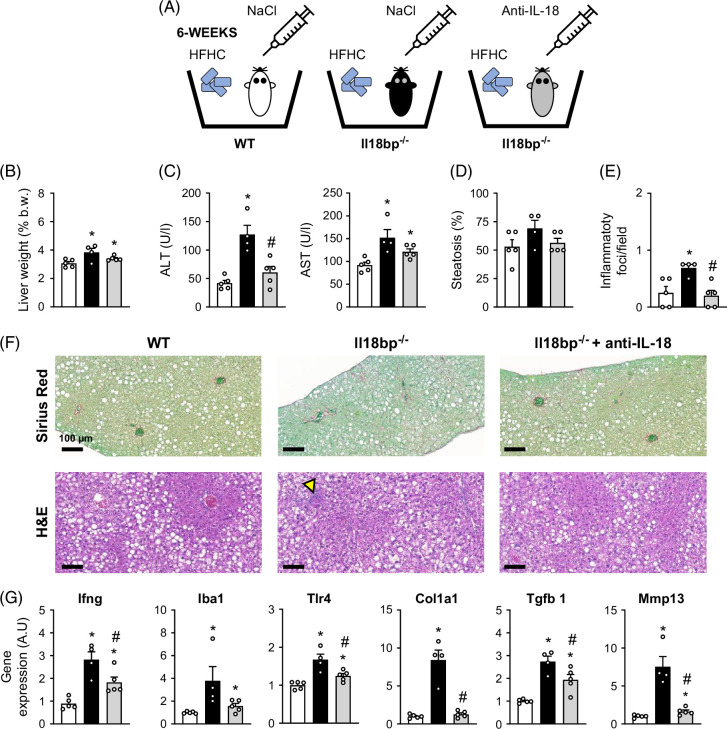
IL-18 neutralization reverses exacerbated inflammation and fibrosis in IL-18BP-deficient mice on HFHC diet. (A) Schematic representation of the study protocol. (B) Relative liver weight. (C) Circulating transaminase levels. (D) Liver steatosis evaluation. (E) Number of inflammatory foci per field (200×). (F) SR and H&E staining of liver sections. Yellow arrows indicate inflammatory foci. (G) Liver gene expression of pro-inflammatory and pro-fibrogenic markers. Bars represent mean±SEM of individual values (circles). *p<0.05 versus WT/NaCl mice and ^#^ *p*<0.05 versus *Il18bp* ^*−/−*^/NaCl mice (Student *t* test), n=4–5 male mice per group. Abbreviations: H&E, hematoxylin–eosin; HFHC, high-fat/high-cholesterol; IL-18, interleukin-18; IL-18BP, interleukin-18 binding protein; SR, Sirius Red; WT, wild type.

**FIGURE 5:**
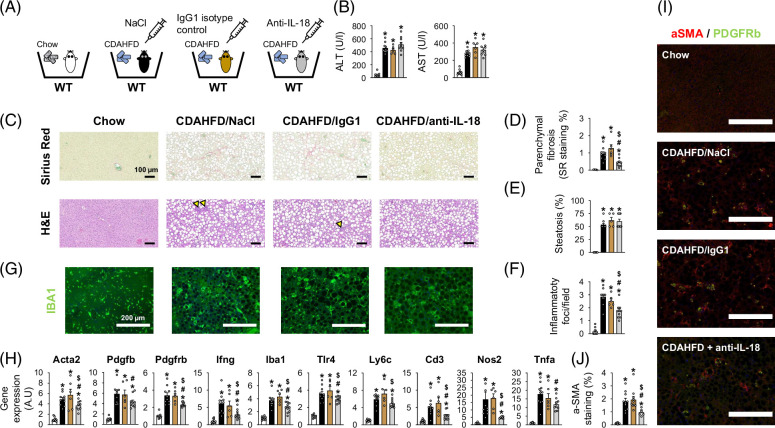
IL-18 neutralization protects against MASLD/MASH in wild-type mice on CDAHFD. (A) Schematic representation of the study protocol. (B) Circulating transaminase levels. (C) SR and H&E staining of liver sections. Yellow arrows indicate inflammatory foci. (D) SR positive staining. (E) Liver steatosis evaluation. (F) Number of inflammatory foci per field (200x). (G) IBA1 immunostaining of liver sections. (H) Liver gene expression of pro-fibrogenic and immune/pro-inflammatory markers. (I) Alpha smooth muscle actin (α*-*SMA)*/*platelet-derived growth factor receptors beta (PDGFRb) immunostaining of liver sections. (J) α*-*SMA positive staining. Bars represent mean±SEM of individual values (circles). *p<0.05 versus chow diet group, ^#^ *p*<0.05 versus CDAHFD-fed NaCl-treated group, and ^$^ *p*<0.05 versus CDAHFD-fed IgG1 isotype-treated group (Student *t* test). n=6–10 male mice per group. Abbreviations: CDAHFD, choline-deficient amino acid-defined high-fat diet; H&E, hematoxylin–eosin; IL-18, interleukin-18; MASH, metabolic dysfunction–associated steatohepatitis; MASLD, metabolic dysfunction–associated steatotic liver disease; SR, Sirius Red; WT, wild type.

First, to check that the endogenous unopposed IL-18 signaling plays a key role in the acceleration of hepatic inflammation and fibrosis observed in IL-18BP–deficient mice on a Western diet, we injected HFHC diet–fed *Il18bp* ^*−/−*^ mice with an anti-IL-18 monoclonal antibody (anti-IL-18 mAb) for 6 weeks. Compared with saline-injected HFHC diet–fed *Il18bp* ^*−/−*^ mice, anti-IL-18–treated HFHC diet–fed *Il18bp* ^*−/−*^ mice exhibited a decrease in ALT levels (Figure 4C), inflammatory foci proportion (Figure 4E), as well as in gene expression of pro-inflammatory (*Ifng*/*Iba1*/*Tlr4*) and pro-fibrotic markers (*Col1a1*/*Tgfb1*/*Mmp13*) (Figure 4G). These results confirm that neutralization of IL-18 limits the excess of hepatic damage, inflammation, and fibrosis initiation observed in IL-18BP–deficient mice on a Western diet. Second, we explored the potential therapeutic value of IL-18 blockade in limiting diet-induced MASLD/MASH progression. To this aim, we fed WT mice a chow or a choline-deficient amino acid-defined high-fat diet (CDAHFD) and injected them with saline or anti-IL-18 mAb. In addition to the saline-injected group, we also injected an IgG1 isotype control to address the potential non-specific effects of antibody injection (Figure 4A). CDAHFD rapidly induces MASLD/MASH (including frank fibrosis) in mice and represents an interesting experimental diet to investigate potential therapeutic intervention within a reasonable time frame.^9^ In fact, we observed that 3 weeks of CDAHFD feeding resulted in much more severe steatosis, inflammation, and fibrosis in WT mice than 14 weeks of HFHC diet-feeding (as illustrated by comparison of WT mice from Figures 2F, 5C), confirming the interest of CDAHFD to rapidly induce severe MASLD/MASH in mice. Compared with saline and IgG1 isotype control-treated mice, WT mice on CDAHFD chronically treated with anti-IL-18 mAb exhibited a reduction in fibrosis (Figures 5C, D) and inflammatory foci proportion (Figure 5F), while their transaminases levels (Figure 5B) and histological steatosis (Figures 5C, E) remained unchanged. Gene expression (Figure 5H), immunostaining (Figures 5I, J) for alpha smooth muscle actin (αSMA) and platelet-derived growth factor beta (PDGFb) and its receptor (PDGFRb) highlighted a limitation of hepatic stellate cells (HSCs) activation in response to the anti-IL-18 mAb administration. In addition, gene expression of a wide panel of inflammatory/immune markers was alleviated in mice treated with the anti-IL-18 mAb (Figure 5H). Altogether, these preclinical results suggest that limitation of IL-18 signaling represents an interesting mechanism to limit MASH progression. Of note, saline-injected and IgG1 isotype control-injected mice did not exhibit any difference on all investigated endpoints, confirming the specificity of the anti-IL-18 effects.

### IFN-gamma deficiency alleviates liver inflammation but not fibrosis in *Il18bp* ^*−/−*^ mice

**FIGURE 6:**
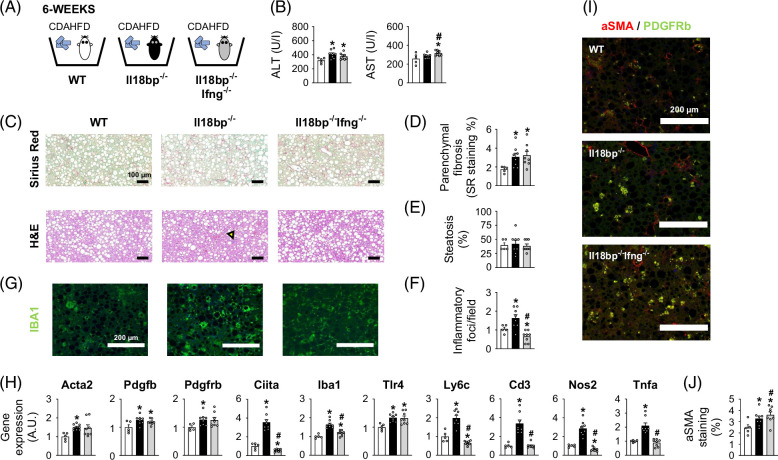
IFN-γ deficiency alleviates inflammation but not fibrosis in IL-18BP–deficient mice on CDAHFD. (A) Schematic representation of the study protocol. (B) Circulating transaminase levels. (C) SR and H&E staining of liver sections. Yellow arrows indicate inflammatory foci. (D) SR positive staining. (E) Liver steatosis evaluation. (F) Number of inflammatory foci per field (200×). (G) IBA1 immunostaining of liver sections. (H) Liver gene expression of pro-fibrogenic and immune/pro-inflammatory markers. (I) Alpha smooth muscle actin (α*-*SMA)/platelet-derived growth factor receptors beta (PDGFRb) immunostaining of liver sections. (J) α*-*SMA positive staining. Bars represent mean±SEM of individual values (circles). *p<0.05 versus WT mice and ^#^ *p*<0.05 versus *Il18bp* ^*−/−*^ mice (Student *t* test). n=5–8 male mice per group. Abbreviations: CDAHFD, choline-deficient amino acid-defined high-fat diet; H&E, hematoxylin–eosin; IBA1, ionized calcium–binding adapter molecule 1; IFN-γ, interferon-gamma; IL-18BP, interleukin-18 binding protein; SR, Sirius Red; WT, wild type.

Molecularly, IL-18 triggers both an IL-1–like intracellular signaling and a specific induction of IFN-γ.^2^,^3^ To delineate IFN-γ–dependent and IFN-γ–independent consequences of unopposed IL-18 signaling in MASH, we generated and studied the double knock-out *Il18bp* ^*−/−*^ *Ifng* ^*−/−*^ mouse. In basal condition (chow diet), *Il18bp* ^*−/−*^ *Ifng* ^*−/−*^ mice exhibited similar liver weight, ALT and AST levels, and liver histology compared with WT and *Il18bp* ^*−/−*^ mice (Supplemental Figures S5B–D, http://links.lww.com/HC9/C166). *Il18* and *Col1a1* were slightly overexpressed while *Tnfa* and *Tlr4* mRNA levels were down expressed in *Il18bp* ^*−/−*^ *Ifng* ^*−/−*^ mice compared with WT or *Il18bp* ^*−/−*^ mice on chow diet (Supplemental Figures S5E, F, http://links.lww.com/HC9/C166). After 6 weeks on CDAHFD (Figure 6A), *Il18bp* ^*−/−*^ *Ifng* ^*−/−*^ mice exhibited similar ALT levels (Figure 6B) and steatosis (Figures 6C, E) as well as similar exacerbation in liver fibrosis (Figures 6C, D) as *Il18bp* ^*−/−*^ mice when compared with WT mice. In line with these findings, activated HSCs markers (*Acta2*, *Pdgfb*, *Pdgfrb*) were similarly expressed between *Il18bp* ^*−/−*^ and *Il18bp* ^*−/−*^ *Ifng* ^*−/−*^ mice (Figures 6H–J). In contrast, *Il18bp* ^*−/−*^ *Ifng* ^*−/−*^ mice presented a reduced hepatic number of inflammatory foci (Figure 6F) as well as a marked reduction in mRNA encoding pro-inflammatory, lymphocytic, and macrophagic markers when compared with *Il18bp* ^*−/−*^ mice (Figure 6H). Together, these results suggest that IFN-γ mediates inflammatory but not pro-fibrotic effects of unopposed IL-18 signaling.

## DISCUSSION

In human patients, it has been reported that rare genetic loss-of-function of IL-18BP results in fulminant hepatitis following a viral infection.^17^ Nevertheless, the role of this protein controlling IL-18 activity remains unknown in the context of metabolic hepatic disorders.

We presently report that IL-18BP is the only member of the IL-1 superfamily to be overexpressed in the liver of a large cohort of patients with MASLD/MASH. Moreover, hepatic IL-18BP expression positively correlates with fibrosis and NAS, revealing a link with the pathology progression. Additional scRNAseq analysis highlighted a main expression of IL-18 and IL-18BP in hepatic macrophages, while IL-18 receptor expression, indirectly reflecting IL-18 cellular targets, is predominant in NK cells, ILC1s, and different T-cell subpopulations, all previously implicated in MASH.^18^,^19^ Interestingly, our present intrahepatic observations deepen previous works reporting elevated circulating levels of IL-18BP in patients with different types of chronic liver diseases,^20^,^21^,^22^ suggesting that IL-18BP could be an interesting serum biomarker to evaluate liver disorders progression.

To functionally implicate IL-18BP in MASLD/MASH, we studied the liver phenotype of IL-18BP-deficient (*Il18bp* ^*−/−*^) mice. In basal condition (chow diet), the liver status of *Il18bp* ^*−/−*^ mice was indistinguishable from that of WT mice, excluding a constitutive hepatic inflammation in accordance with the absence of spontaneous systemic inflammation previously reported in this mouse line.^7^ In contrast, on HFHC and CDAHFD diets, classically used to induce MASLD/MASH in rodents, we observed that *Il18bp* ^*−/−*^ mice present worsened hepatic inflammation compared with WT mice, suggesting that IL-18BP represents an important gatekeeper of liver integrity during MASLD/MASH. In fact, IL-18BP forms high-affinity complexes with IL-18, thus blocking IL-18/IL-18 receptor interactions and signaling.^2^,^3^ Moreover, binding affinity of IL-18 with IL-18BP is much higher than to its IL-18 receptor, and the high concentrations of IL-18BP leaves only very few free (and thus bioactive) IL-18 molecules in basal conditions.^2^,^3^ In contrast, in *Il18bp* ^*−/−*^ mice, all IL-18 produced is present as free, that is unbound to IL-18BP, in the blood and tissues. Similarly to the action of administered IL-18,^2^,^3^ the endogenous unopposed IL-18 signaling led to a consistent hepatic overexpression of pro-inflammatory cytokines (*Il1b*, *Il12*, *Tnfa*, *Ifng*) and chemokines (*Ccl2*, *Cxcl2*, *Cxcl9*, *Cxcl10*) driving type 1 inflammation in diet-challenged *Il18bp* ^*−/−*^ mice. Mechanistically, immune-blockade of free IL-18 allowed for a drastic reduction in hepatic damage and pro-inflammatory status of *Il18bp^−/−^* mice, directly involving IL-18 over-signaling in MASH progression. Of note, exacerbated hepatic inflammation observed in diet-challenged *Il18bp* ^*−/−*^ mice occurred independently of steatosis (which remained similar to that of WT mice), indicating that MASH progression is uncoupled from levels of hepatic fat storage in this genetic context.

Liver fibrosis is associated with a higher risk of mortality in patients with MASLD/MASH,^1^ representing an important biological mechanism to inhibit in order to prevent disease progression. A major finding of our study is the consistent increase in fibrosis observed in the liver of nutritionally challenged *Il18bp* ^*−/−*^ mice compared with WT mice. On the contrary, blockade of free IL-18 with a monoclonal antibody reduces diet-induced fibrosis in WT mice, opening therapeutic perspectives. Taken together, these results implicate IL-18 signaling as an important mediator of diet-induced liver fibrosis and IL-18BP as a key endogenous modulator of this process. Previous studies have shown a role for IL-18 in heart, lung, or kidney fibrosis.^23^ In addition, the overactivity of the NLRP3 inflammasome activates HSCs that produce collagen, while an inflammasome component deficiency limits hepatic collagen deposition.^24^ Recently, it has also been shown that IL-18 receptor-deficient mice are protected against hepatic fibrosis.^25^ However, molecular and cellular mechanisms underlying fibrosis downstream of IL-18 signaling remain elusive. It has been reported that excessive IL-18 signaling exacerbates liver injury through NK and T cell-mediated IFN-γ production.^26^ In line, DGE and GSEA in our RNAseq experiments confirmed that transcripts significantly upregulated in *Il18bp* ^*−/−*^ mice livers were enriched for gene ontology clusters related to IFN-γ response and signaling. In this context, we generated and nutritionally challenged a double knock-out *Il18bp* ^*−/−*^ *Ifng* ^*−/−*^ mouse line, harboring an excessive endogenous IL-18 signaling, in the absence of downstream IFN-γ signaling, in order to delineate IFN-γ-dependent and IFN-γ-independent consequences of unopposed IL-18 signaling in MASLD/MASH. Following a nutritional challenge, *Il18bp* ^*−/−*^ *Ifng* ^*−/−*^ mice exhibited a full abrogation of hepatic inflammation but the same aggravated fibrosis as *Il18bp* ^*−/−*^ mice when compared with WT mice. These results demonstrate that IFN-γ mediates inflammation but not fibrosis downstream of excessive IL-18 signaling, and that fibrosis is not a simple resultant of exacerbated inflammation in *Il18bp* ^*−/−*^ mice.

We also observed a decreased NK cell content in the liver of nutritionally challenged *Il18bp* ^*−/−*^ mice, corroborating the splenic depletion of mature NK cells previously observed in this mouse line.^27^ Previous works have shown that NK T cell depletion by dietary fatty acids contribute to MASH worsening,^28^ and that intrahepatic NK cells seems to play a protective role against the development of fibrosis,^29^ possibly by regulating liver macrophages polarization^30^ and exerting a cytotoxic activity toward HSCs.^31^ Other works report that NK T cells may have a protective role at the early stage of MASH, while they could act as a progression factor at a more advanced stage of MASH progression.^32^,^33^,^34^

Other immune mechanisms involved in fibrosis include M2-macrophage differentiation, infiltration, and survival.^19^ In the absence of hepatic transcriptional changes regarding the most classical M2-macrophage markers (*Il4*, *Arg1*, and *Cd206*), we observed higher hepatic levels of *Il5*, *Il10*, and *Il13* mRNAs in *Il18bp* ^*−/−*^ versus WT mice. Moreover, hepatic *Il10* and *Il13* mRNAs positively correlated with collagen content in *Il18bp* ^*−/−*^ and WT mice. These results are in accordance with previous works implicating this set of anti-inflammatory cytokines in different liver fibrosis contexts. In fact, *Il5* ^*−/−*^ mice exhibit less hepatic fibrosis following parasite infection,^35^ while IL-13 appears to drive an alternative TGF-β–independent pro-fibrotic pathway in MASH.^36^

Further experiments involving dedicated FACS analysis and snRNAseq approaches are now needed to elucidate the role of NK cells and macrophages subpopulations in the pro-fibrotic phenotype of *Il18bp* ^*−/−*^ mice livers.

The cellular source of collagen accumulation in nutritionally challenged *Il18bp* ^*−/−*^ mice remains an open question. In chemically induced liver fibrosis, IL-18 receptor (*Il18r1*) was found to be highly expressed in a subset of activated HSCs, but not in quiescent HSCs.^25^ In this context, IL-18BP can block the self-induced IL-18 signaling and potently inhibit the activation of HSCs and resulting fibrosis. Our scRNAseq analysis based on the Liver Cell Atlas revealed that IL-18 receptor is mainly expressed by fibroblasts featured as mesothelial cells, not by HSCs. Previous studies using conditional cell lineage analysis have shown that mesothelial cells undergo mesothelial–mesenchymal transition and give rise to HSCs and myofibroblasts during liver fibrogenesis.^37^ Moreover, RNAseq analysis reveals that IL-18BP–deficient mice present, in addition to abundant fibrillar type I collagen overexpression, an enrichment in type IV (Col4a1, Col4a2) collagen expression, which are key components of basement membranes and sheet-like scaffold at the basal site of epithelia and endothelia. Further investigations are nevertheless needed to better understand which collagen-producing cell precursors and which stage of their activation are stimulated by IL-18 signaling.

Growing evidence associates defects in cholesterol homeostasis and MASH. Compared with WT mice, livers from HFHC diet–fed *Il18bp* ^*−/−*^ mice exhibited a striking down-expression of enzymes gating both the classical and the alternative pathway of bile acids synthesis (*Cyp8b1*, *Cyp7b1*, *Hsd3b7*). Similarly, *Il1ra* ^*−/−*^ mice (deficient for the IL-1 receptor antagonist, thus harboring an unopposed IL-1 signaling) presented hepatic downregulation of *Cyp7a1* (the rate-limiting enzyme in bile acid synthesis) on HFHC diet.^38^ Taken together, these results demonstrate that endogenous inhibitors of the IL-1 superfamily (IL-18BP, IL-1Ra) are required to maintain normal expression of bile acids synthesis enzymes and avoid exacerbated negative feedback on bile acids production in conditions of cholesterol/cholate overload. Concomitantly, we observed an upregulation of cholesterol 25-hydroxylase (*Ch25h*), the monooxygenase catalyzing the conversion of cholesterol to 25-hydroxycholesterol (25-HC), in *Il18bp* ^*−/−*^ mice. CH25H is a target of interferon-γ, and 25-HC presents well-described anti-viral properties.^39^ However, 25-HC potently stimulates an inflammatory response in activating NF-κB and the secretion of pro-inflammatory cytokines by hepatic macrophages.^40^,^41^ Therefore, an unbalanced hepatic production of oxysterols at the expense of bile acids could contribute to MASH progression in *Il18bp* ^*−/−*^ mice. This phenomenon could be amplified by downregulation of *Cyp7b1* observed in *Il18bp* ^*−/−*^ versus WT mice, not only on HFHC diet, but also on other nutritional stresses leading to MASLD/MASH (data not shown). *Cyp7b1* controls the levels of intracellular oxysterols, converting 25-HC into 7α-hydroxylated oxysterols that enter BA synthesis pathways. Previous works have shown that *Cyp7b1* ^*−/−*^ mice accumulated 25-HC in the liver,^40^,^41^ and that CYP7B1 loss-of-function in human leads to severe cholestasis/cirrhosis in early life.^42^ Moreover, CYP7B1 is also inhibited in MASH patients, inversely correlating with liver injury markers.^43^

In conclusion, our experimental and clinical observations reveal a previously unknown role for IL-18BP as a gatekeeper of liver integrity on a Western diet, in particular regarding inflammation and fibrosis. Further work involving hematopoietic-specific *Il18* ^*−/−*^/*Il18r1* ^*−/−*^/*Il18bp* ^*−/−*^ mice is now required to better evaluate the intrahepatic contribution of IL-18BP in MASH progression. Increasing IL-18BP amount or activity (a therapeutic option already clinically validated to treat rare systemic auto-inflammatory diseases^44^) represents interesting pharmacological perspectives to treat MASLD/MASH.

## Supplementary Material
