# Supplementary material for: Interleukin-18 binding protein protects against metabolic steatohepatitis
Source: Hepatol Commun. 2025 Nov 20;9(12):e0840. doi: 10.1097/HC9.0000000000000840 (PMC12614690; doi:10.1097/HC9.0000000000000840)

Supplemental Figure 1. Cell-specific expression of *IL18, IL18BP* and *IL18R1* in human liver.

A) Umap projection of *IL18*, *IL18BP* and *IL18R1* in whole cell types composing human liver. B) Umap projection of *IL18* and *IL18BP* in human hepatic myeloid cells. C) Umap projection of *IL18R1* in human hepatic lymphoid cells. Raw data originates from the liver cell atlas (https://www.livercellatlas.org).

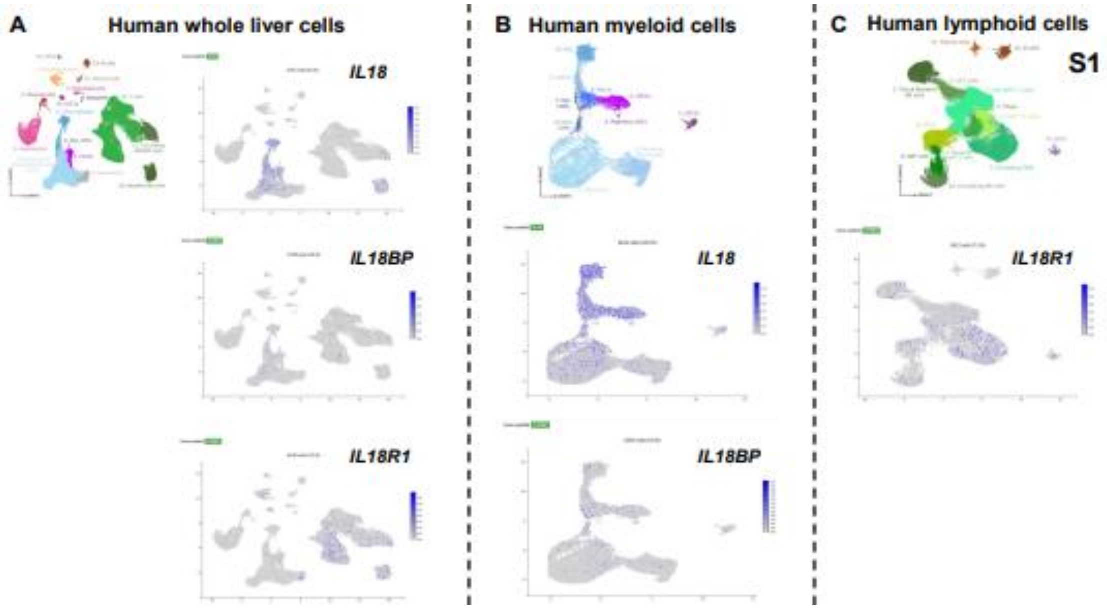

Supplemental Figure 2. Cell-specific expression of *Il18*, *Il18bp* and *Il18r1* in standard and MASLD mice liver.

(A) UMAP projection of *Il18*, *Il18bp* and *Il18r1* expression in all liver cell populations in standard mice. (B) UMAP projection of *Il18*, *Il18bp* and *Il18r1* expression in all liver cell populations in MASLD (HFD-fed) mice. Raw data originates from the liver cell atlas (https://www.livercellatlas.org).

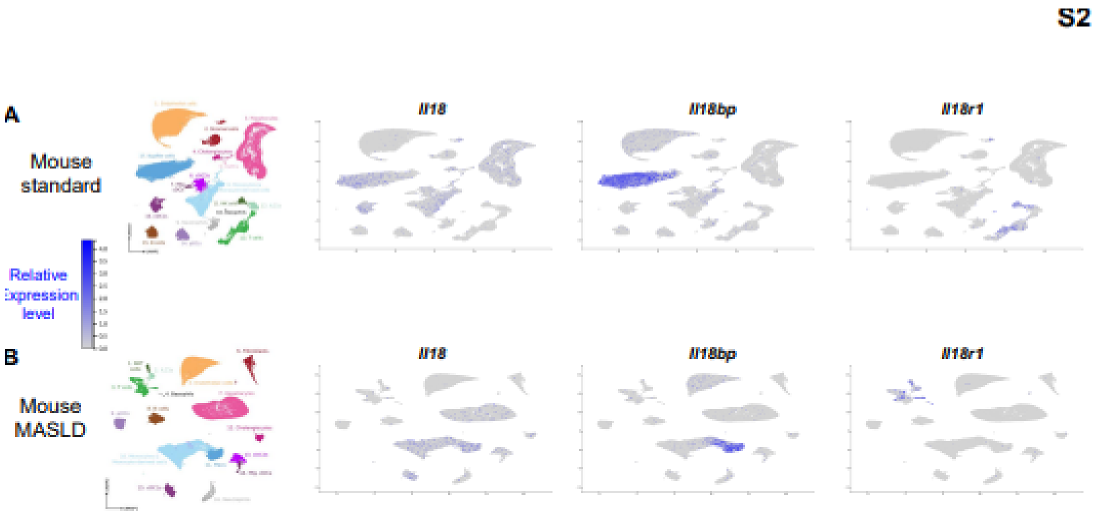

Supplemental Figure 3. Cell-specific expression of *Il18* and *Il18bp* in liver myeloid compartment in standard and MASLD mice liver.

(A) UMAP projection of *Il18* and *Il18bp* in liver meloid cell populations in standard mice liver. (B) UMAP projection of *Il18* and *Il18bp* in liver meloid cell populations in MASLD mice liver. Raw data originates from the liver cell atlas (https://www.livercellatlas.org).

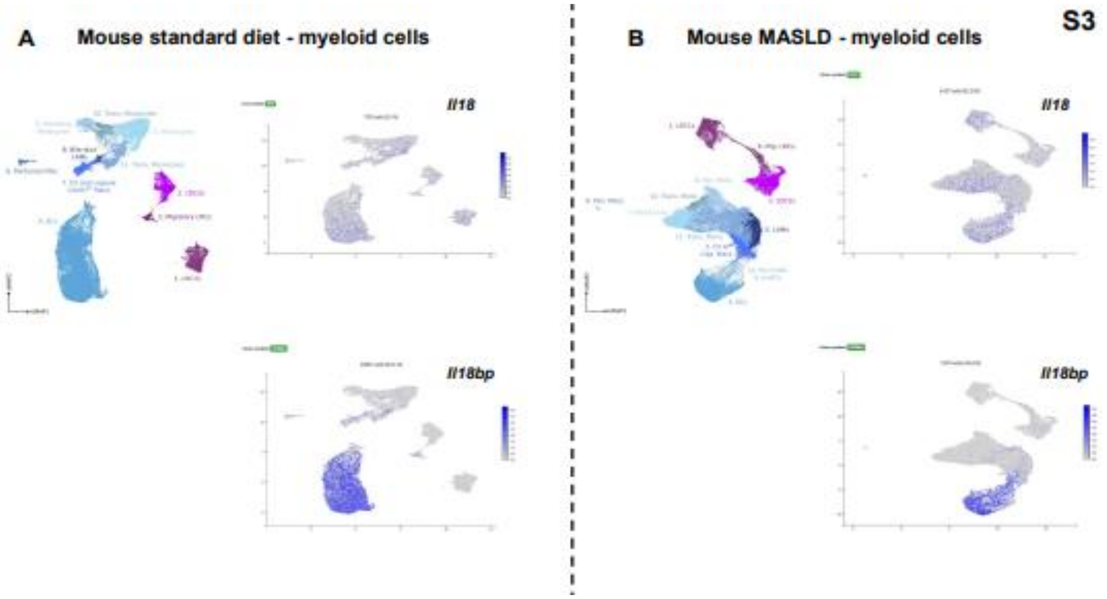

Supplemental Figure 4. Cell-specific expression of *Il18r1* in liver lymphoid and fibroblast compartment in standard and MASLD mice liver.

(A) UMAP projection of *Il18r1* in liver lymphoid and fibroblast cell populations in standard mice liver. (B) UMAP projection of *Il18r1* in liver lymphoid and fibroblast cell populations in MASLD mice liver. Raw data originates from the liver cell atlas (https://www.livercellatlas.org).

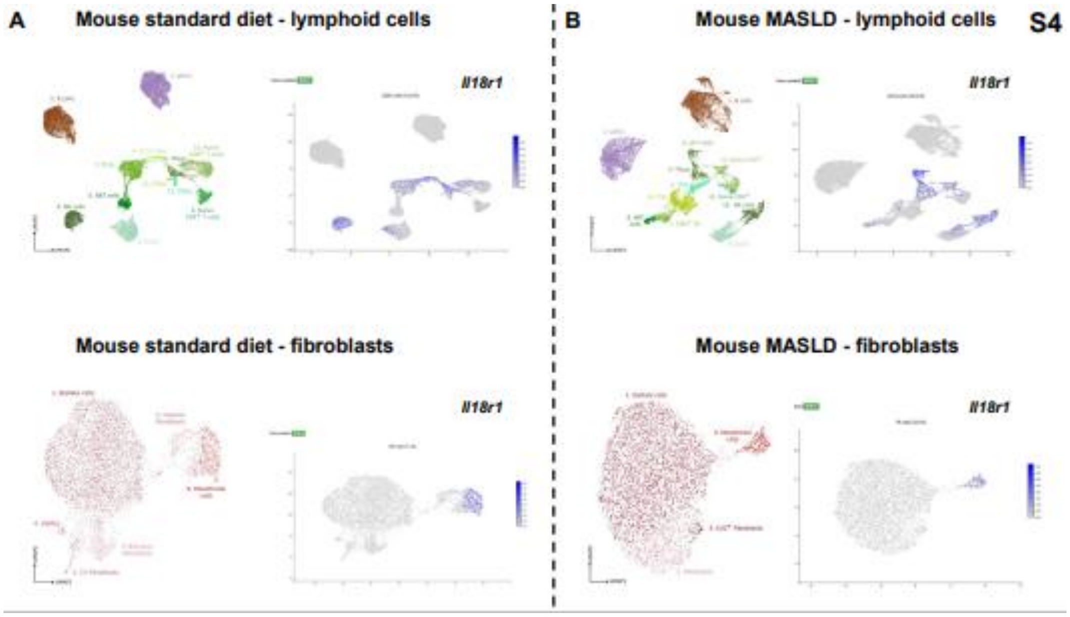

Supplemental Figure 5. IL-18BP deficiency and c*oncomitant IL-18BP/IFN-γ deficiency* do not induce liver alteration in basal conditions (chow diet).

(A) Schematic representation of the study protocol. (B) Relative liver weight (expressed as percentage of body weight). (C) Circulating transaminases levels. (D) SR and *H&E staining of liver sections.* (E) Liver gene expression of pro-inflammatory markers. (F) Liver gene expression of pro-fibrogenic markers. Bars represent mean ± SEM of individual values (circles). Levels of significance: *$p < 0.05$ vs. WT mice and $^{\#}p < 0.05$ vs. *Il18bp*$^{-/-}$ mice (*Student's t-test*). n = 6–8 *male* mice per group.

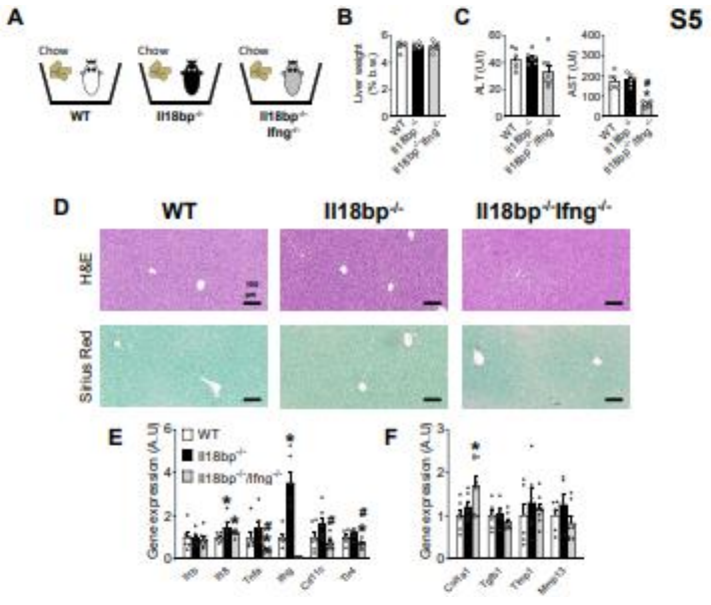

Supplement: Supplementary file 1 [file hc9-9-e0840-s001.pdf]
