# Supplementary material for: Interleukin-18 binding protein protects against metabolic steatohepatitis
Source: Hepatol Commun. 2025 Nov 20;9(12):e0840. doi: 10.1097/HC9.0000000000000840 (PMC12614690; doi:10.1097/HC9.0000000000000840)
Supplement: Supplementary file 2 [file hc9-9-e0840-s002.docx]

Supplemental table 1.
